# Corrosion Properties of S-Phase/Cr_2_N Composite Coatings Deposited on Austenitic Stainless Steel

**DOI:** 10.3390/ma15010266

**Published:** 2021-12-30

**Authors:** Sebastian Fryska, Jolanta Baranowska

**Affiliations:** Department of Materials Technology, West Pomeranian University of Technology, Piastow Av. 19, 70-310 Szczecin, Poland; Jolanta.Baranowska@zut.edu.pl

**Keywords:** S-phase, Cr_2_N chromium nitride, composite coatings, reactive magnetron sputtering, corrosion resistance, austenitic stainless steel

## Abstract

In order to study the suitability of the S-phase layers as the interlayer for Cr_2_N chromium nitride coatings, a number of composite coatings were deposited by the reactive magnetron sputtering (RMS) method on austenitic steel substrates with various initial surface conditions (as delivered and polished) and their corrosion resistance was assessed. Coatings with S-phase interlayer were deposited at three different nitrogen contents in the working atmosphere (15%, 30%, and 50%), which influenced the nitrogen concentration in the S-phase. Coatings with chromium, as a traditional interlayer to improve adhesion, and uncoated austenitic stainless steel were used as reference materials. Detailed microstructural and phase composition studies of the coatings were carried out by means of scanning electron microscopy (SEM), optical microscopy (LM), and X-ray diffraction (XRD) and were discussed in the context of results of corrosion tests carried out with the use of the potentiodynamic polarization method conducted in a 3% aqueous solution of sodium chloride (NaCl). The performed tests showed that the electrochemical potential of the S-phase/Cr_2_N composite coatings is similar to that of Cr/Cr_2_N coatings. It was also observed that the increase in the nitrogen content in the S-phase interlayer causes an increase in the polarization resistance of the S-phase/Cr_2_N composite coating. Moreover, with a higher nitrogen content in the S-phase interlayer, the polarization resistance of the S-phase/Cr_2_N coating is higher than for the Cr/Cr_2_N reference coating. All the produced composite coatings showed better corrosion properties in relation to the uncoated austenitic stainless steel.

## 1. Introduction

Hard ceramic coatings produced by physical vapour deposition (PVD) methods, including reactive magnetron sputtering (RMS) methods, such as chromium nitrides or titanium nitrides, are commonly used as protective coatings in cutting tools, injection molds, and injection nozzles for plastics, as well as for decorative coatings or electrodes in fuel cells [1,2,3,4,5,6,7,8,9,10]. Such a wide range of applications stems from their high hardness and wear resistance, as well as high corrosion resistance [11,12,13,14,15,16,17,18,19,20,21,22,23]. The functional properties of these coatings are determined by the number of defects in the coating and the adhesion of the coating to the substrate. Many studies attempt to determine the influence of the initial state of the substrate and the parameters of the deposition process on the number and type of defects of the coating and its functional properties. It has been proven that reducing the number of defects in the coating, such as pores, voids, inclusions, or droplets, increases the resistance of the coating to pitting and crevice corrosion [24,25,26,27]. Various types of interlayers and multilayer coatings have been proposed as methods of reducing the number of defects in coatings and increasing the material density, which reduces the penetration of corrosive agents through the coating [12,23,28,29,30,31,32,33].

A separate problem is the significant difference in hardness between the coated substrate and the deposited coating, which reduces the adhesion of the coating to the substrate, and thus facilitates the penetration of corrosive agents through the coating into the material. This applies in particular to substrates made of soft metals, such as austenitic stainless steels or non-ferrous materials. For this reason, in order to improve the functional and corrosive properties, various types of interlayers are used, most often chromium [1,2,3,4,5,6,7,8,9,10,11,12,13,14,15,16,17,18,19,20,21,22,23,24,25,26,27,28,29,30,31,32,33], less often titanium or nickel [25,28,29].

In the case of austenitic stainless steel, which is a widely used material, mainly due to its very high corrosion resistance, its low hardness can be improved by producing an S-phase based on it. The S-phase is a supersaturated solid solution of nitrogen in the austenitic structure. It can be produced in austenitic stainless steel, but also in other materials with an austenitic structure [34]. The S-phase can be produced as a layer on the surface of the workpiece by gas or plasma nitriding, but also as a coating using reactive deposition by PVD methods. The hardness of the S-phase can be up to 20 GPa, while retaining very good corrosion resistance, comparable to austenitic stainless steel [34,35,36,37,38,39,40,41,42,43]. However, in some applications, the performance of the S-phase may be insufficient, whereby the use of another ceramic coating as an outer coating, e.g., chromium nitride, can further improve the properties of the austenitic stainless steel.

As mentioned earlier, various types of interlayers, most often a chromium layer, are used to improve the performance of materials by depositing protective coatings on their surface. Replacing the chromium interlayer with the S-phase interlayer may improve the properties of the coating deposited on an austenitic stainless steel surface for several reasons. First, the S-phase is characterized by a higher hardness compared to the chromium layer, which has the hardness of about 11 GPa [30]. Moreover, previous studies have shown that it is possible to create a nitrogen diffusion layer in the coated substrate under the coating [35,37]. This allows to reduce the internal stresses in the coating by decreasing the difference in hardness between the coating and the coated substrate, and in this way to improve the adhesion of the coating to the substrate. Fryska et al. [44] report that the use of an S-phase as an interlayer during the deposition of a Cr_2_N coating improves its adhesion to austenitic stainless steel substrates. It is also possible to increase the hardness of the AISI 304 substrate-Cr_2_N coating system using the S-phase as an interlayer, since the S-phase, showing properties intermediate between the substrate and the outer coating, supports the latter. Cost reduction can be an additional factor, as the targets made of austenitic stainless steel are much cheaper compared to the chromium ones used for magnetron sputtering.

However, the influence of the S-phase as an interlayer on the corrosion resistance of the AISI 304 austenitic steel-Cr_2_N coating system has not been described so far. The paper presents the results of studies on the corrosion properties of austenitic stainless steel covered with composite coatings made of chromium nitride (Cr_2_N) as an outer coating and S-phase as an interlayer used to replace the chromium interlayer.

## 2. Materials and Methods

The coatings were deposited using a laboratory set-up for producing coatings by the reactive sputtering method (RMS) (Orion 5, AJA International, Scituate, MA, USA). There are three magnetron guns in the working chamber, each equipped with a 750 W DC power supply. The coatings were produced by sputtering the targets in the form of discs with the diameter of 5.1 cm made of austenitic stainless steel (AISI 304) or chromium (Cr 99.99 %). The chemical composition (mass %) of the steel used was as follows: C < 0.05%, Cr—19%, Ni—9%, Fe—balans. In each deposition process, two austenitic stainless steel substrates (also AISI 304) were placed in the chamber. The substrates had different initial states. One substrate was cut directly from the sheet in as delivery state (DS—mechanically polished sheet). The second substrate was cut, then ground with sandpaper and polished with diamond polishing pastes and with the use of an aqueous suspension of aluminum oxide (P—polished). The substrates prepared in this way were characterized by a roughness of R_a_ respectively: 0.06 μm for delivery state and 0.02 μm after polishing. The roughness of the substrates before and after the polishing process was measured with a Dektak 6M mechanical profilometer (Dektak 6M, Bruker Corporation, Billerica, MA, USA). Subsequently, the substrates were placed in the working chamber on a heated, rotating table. The temperature of the substrates was 400 °C during the entire deposition process. After placing the substrates in the working chamber, the substrates were plasma cleaned before initiating the coating deposition process. Plasma cleaning was carried out by polarizing the table with a voltage with radio frequency (RF) of about −200 V for 30 min. After the cleaning process was completed, the polarization voltage of the table was reduced to about −50 V and maintained at this level throughout the deposition of the coating. The plasma cleaning process of substrates was carried out with the use of an inert gas, argon (100% Ar). However, during the deposition of the coatings, argon (Ar) or a mixture of gases, namely argon (Ar) and nitrogen (N_2_), was used. The total working pressure of the gases during the plasma cleaning was 2.67 Pa (20 mTorr), while during the deposition of the coatings it was 0.53 Pa (4 mTorr).

**Figure 1:**
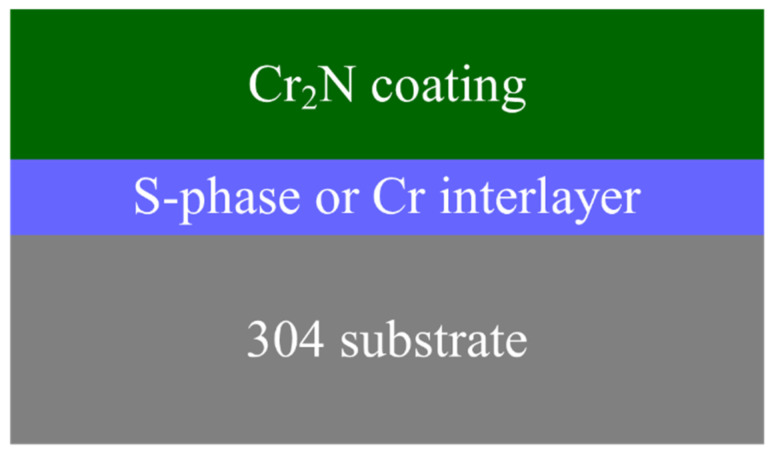
Schematic structure of composite coating deposited in experiments [44].

S-phase/Cr_2_N composite coatings were produced as follows: an interlayer of S-phase was deposited on a cleaned substrate, whereby the share of nitrogen in the working atmosphere was 15%, 30% or 50% vol. After 10 min of depositing the interlayer, the magnetron gun with the AISI 304 steel was turned off and the two magnetron guns equipped with chromium targets were turned on. To obtain a coating composed of chromium nitride Cr_2_N, the mixture of working gases consisted of 70% vol. argon and 30% vol. nitrogen. The deposition time of the Cr_2_N coating was 60 min. Other process parameters, such as the substrate temperature or the total pressure of the working gases, did not change. Cr/Cr_2_N coating with a chromium interlayer was used in the tests as a reference system for S-phase/Cr_2_N composite coatings. The chromium interlayer was obtained by sputtering the chromium source for 10 min under argon. After this time, the Cr_2_N coating was deposited in the same way as for S-phase/Cr_2_N composite coatings. The schematic structure of the composite coatings is shown in Figure 1. All coating deposition conditions are summarized in Table 1.

The phase composition of the coatings was investigated using the X-ray diffraction method with the use of CuKα radiation (X’PERT PANalytical, Almelo, The Netherlands). During the tests, the Bragg–Brentano geometry in the angular range of 35–80° 2theta was used. The morphology of the coatings and their microstructure were examined using a scanning electron microscope (Hitachi SU-70, Tokio, Japan) and an optical microscope (Nikon MM-40, Tokio, Japan). The corrosion properties of the coatings were tested using the potentiodynamic polarization method in a 3% aqueous NaCl solution. Two measurements were made on each of the tested samples. The ATLAS 9833 electrochemical interface (ATLAS-SOLLICH, Rębiechowo, Poland) was used for the process of polarization of the tested samples in the range from −1500 mV to +1500 mV. The speed of the voltage change (sweep ramp) was 5 mV/s. The exposure area of the sample was 0.283 cm^2^. A calomel electrode and a platinum electrode were used as a reference and auxiliary electrode, respectively. The Tafel method was used to analyse the obtained potentiodynamic curves.

## 3. Results and Discussion

**Figure 2:**
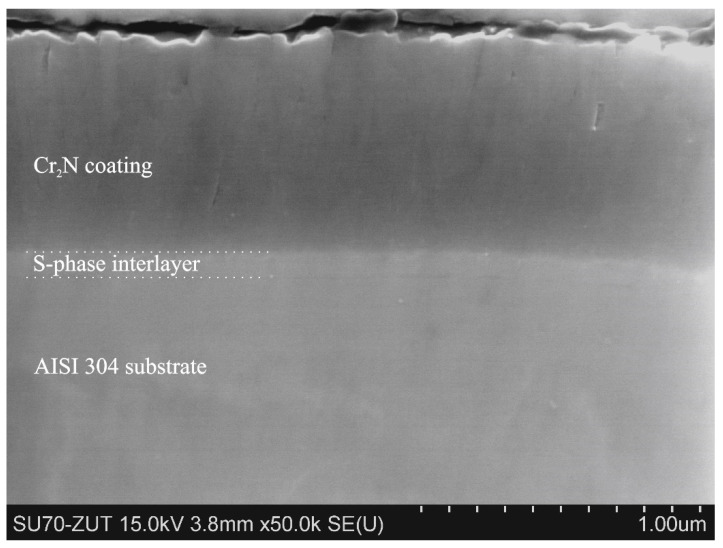
Cross-section of S-phase (30 vol.% of N_2_)/Cr_2_N coating deposited on substrate in delivery state, SEM.

All the deposited coatings were about 860 nm thick, with an interlayer thickness of about 60 nm. The coatings were compact, made of fine columnar grains with a few visible small voids between them, as shown in Figure 2.

**Figure 3:**
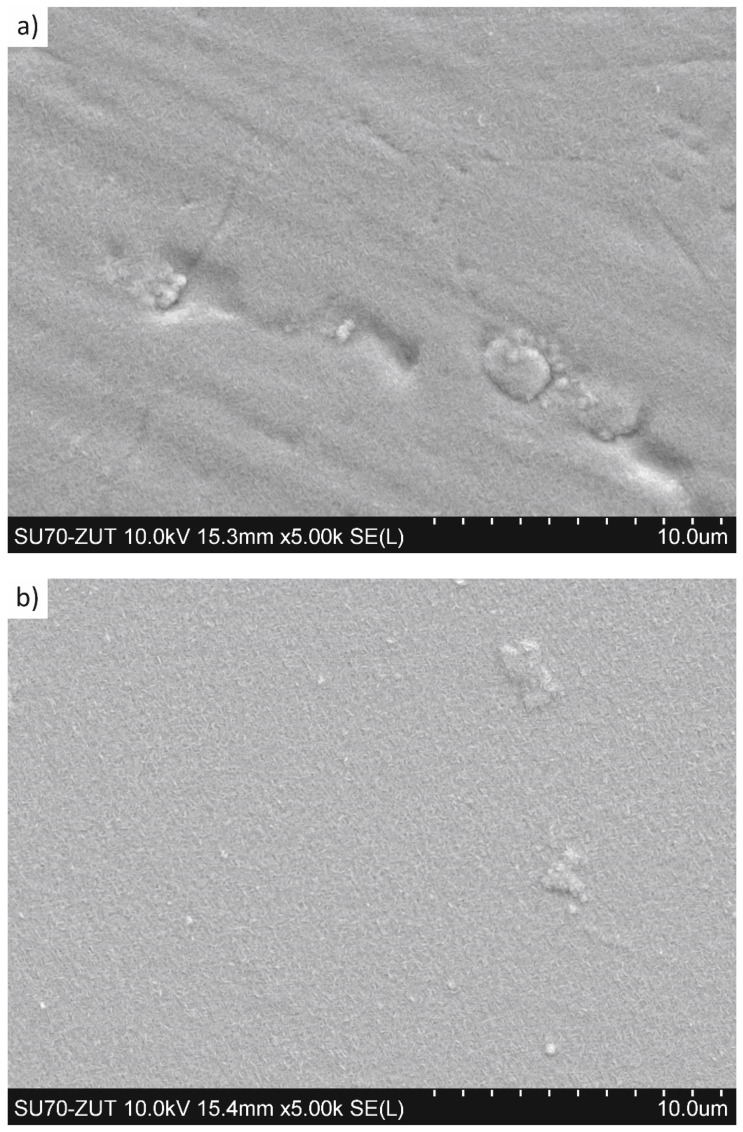
To surface view of S-phase (50 vol.% of N_2_)/Cr_2_N coating deposited on: (**a**) substrate in delivery state, (**b**) polished substrate, SEM.

Figure 3 shows the morphology of coatings deposited on the substrates with different initial states. The coatings deposited on the substrate in delivery state were characterized by many defects, such as grooves, pores, or discontinuities (pinholes), as well as clearly marked boundaries of austenite grains from the substrate (Figure 3a). These defects did not occur in coatings deposited on polished substrates. Here, however, other types of coating defects were observed, such as inclusions (Figure 3b). Moreover, in the case of coatings deposited on polished substrates, the boundaries of austenite grains from the substrate were visible, but to a much lesser extent than in the case with the substrates in delivery state.

**Figure 4:**
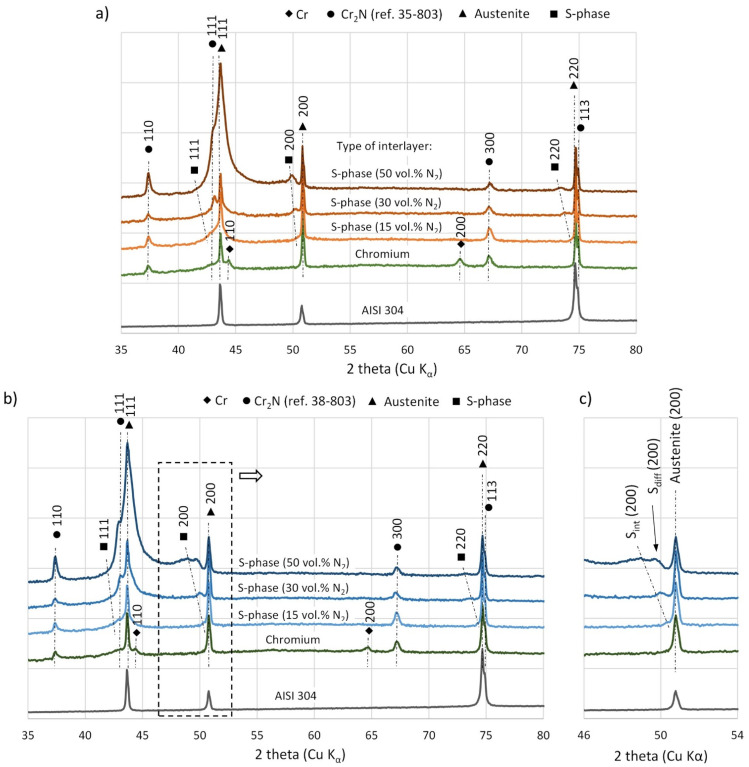
Diffraction patterns of the composite coatings deposited on: (**a**) as delivered substrates, (**b**) polished substrates, (**c**) diffraction peaks for crystallographic planes (200) marked on (**b**); XRD Cu Kα; S_int_—S-phase interlayer, S_diff_—S-phase diffusion layer.

XRD analysis of diffraction patterns for all deposited composite coatings confirmed the presence of the outer Cr_2_N coating (Figure 4). Moreover, depending on the interlayer used, chromium or S-phase diffraction peaks were also observed. The S-phase is a metastable supersaturated solution of nitrogen in the austenitic structure. Therefore, the peaks from this phase are observed near the peaks originating from the austenite. However, they are shifted towards the smaller 2theta angles due to the higher lattice parameter of this phase. As the latter depends on the nitrogen content in the S-phase, the size of this shift also correlates with the nitrogen content, i.e., the greater the nitrogen content, the greater the shift of the peaks from the S-phase towards the smaller 2theta angles.

No significant differences were observed in the phase composition of coatings deposited on substrates with different initial states. The only exception was the phase composition of the coating deposited on the polished substrate, where a coating made from S-phase was deposited as an interlayer at 50% vol. of nitrogen in the working atmosphere. In this case, an additional peak was observed, visible in Figure 4c for the group of crystallographic planes (200). It represents the S-phase that, however, originated not from the interlayer, but from the diffusion layer formed in the substrate during coating deposition. The resulting diffusion layer is in fact also an S-phase, but with a reduced nitrogen content compared to that in the deposited interlayer. Hence, a smaller shift in the diffraction peak compared to the interlayer is observed. The formation of a nitrogen diffusion layer in the austenitic substrate during the deposition of the S-phase coatings was observed in previous studies even at a temperature of 350 °C [35].

Moreover, it was observed that the increase in nitrogen content in the working atmosphere during the deposition of the S-phase interlayer caused an increase in the intensity of diffraction peaks both from the S-phase itself and from the Cr_2_N coating. This effect may be related to the size of the grains in the deposited coating. When the nitrogen content in the working atmosphere is low, the S-phase coating is characterized by a fine-grained structure, while in a high nitrogen atmosphere, the deposited coating is made of much larger grains [37]. This could be due to the fact that the grain size of the S-phase interlayer impacts the nucleation process of the Cr_2_N coating with smaller or larger grains, depending on whether the S-phase interlayer is composed of smaller or larger grains, respectively. This effect can be used to control the grain size of the coating, where a fine-grained structure can increase its density and in this way may improve the corrosion resistance of the coating [13,26,32].

**Figure 5:**
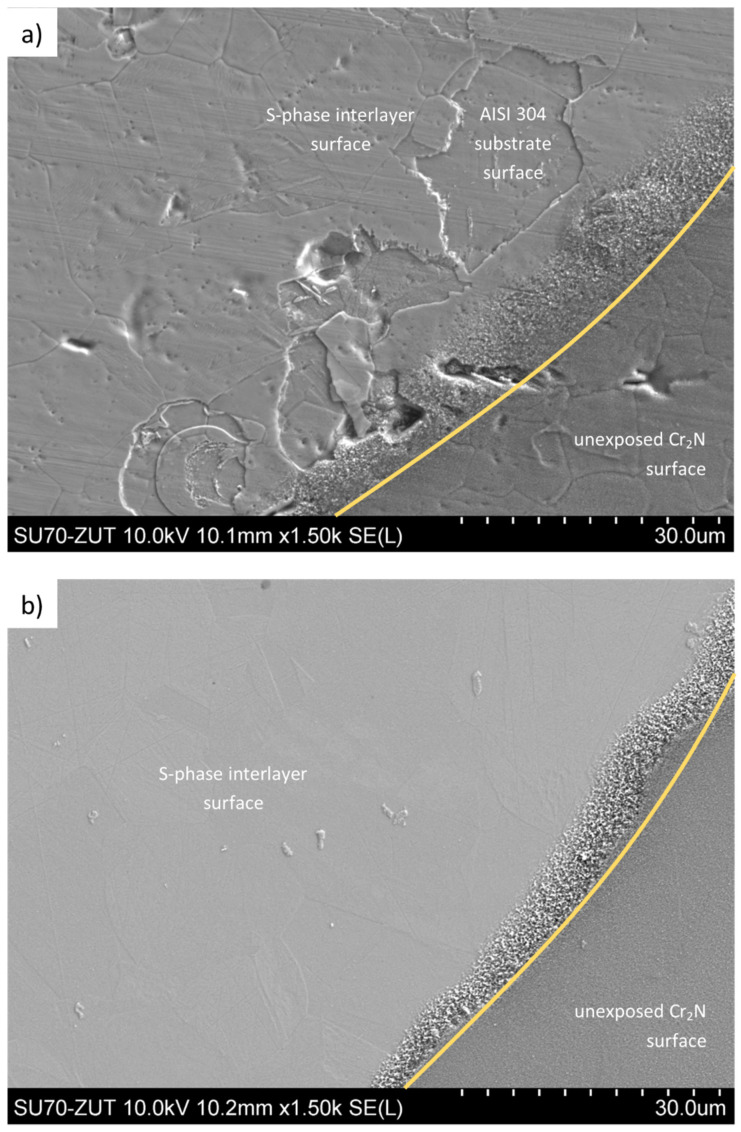

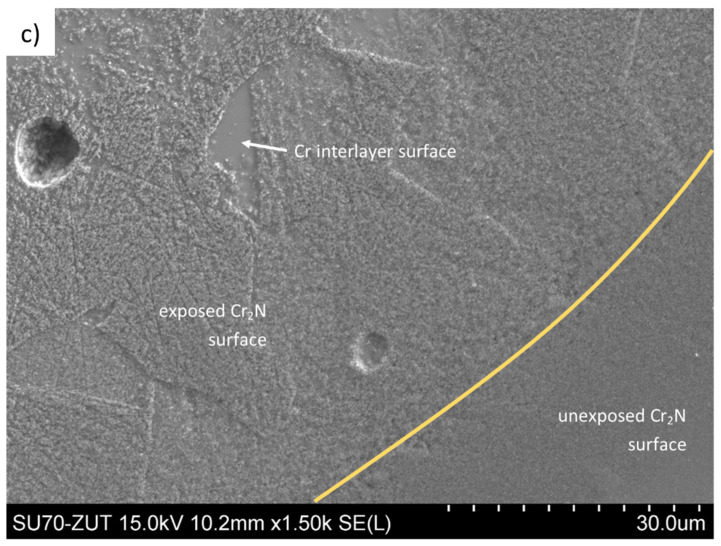
Top surface of coating: (**a**) S-phase (50 vol.% of N_2_)/Cr_2_N (substrate in as delivery state), (**b**) S-phase (50 vol.% of N_2_)/Cr_2_N (polished substrate) and (**c**) Cr/Cr_2_N (substrate in as delivery state) after potentiodynamic corrosion test, SEM.

**Figure 6:**
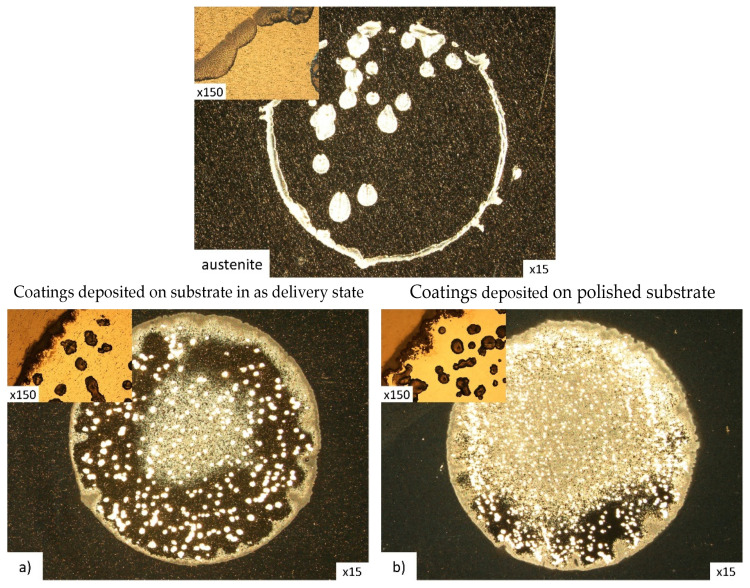

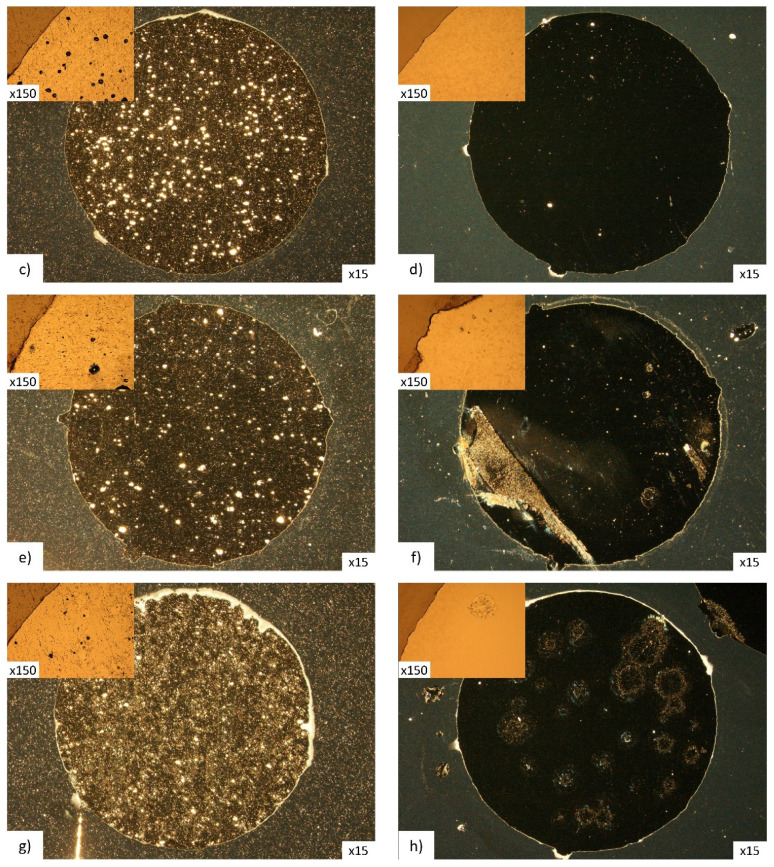
Coating surface after corrosion tests; coatings deposited on substrates in as delivery state (left column) and on polished ones (right column); (**a**,**b**)—Cr interlayer; (**c**,**d**)—S-phase (15 vol.% of N_2_) interlayer; (**e**,**f**)—S-phase (30 vol.% of N_2_) interlayer; (**g**,**h**)—S-phase (50 vol.% of N_2_) interlayer (light microscopy).

Figure 5 and Figure 6 show the surfaces of composite coatings deposited on substrates with various initial states, after corrosion tests. During the corrosion resistance measurements, coating defects, i.e., grooves, pores, or pinholes, resulted from pitting and crevice corrosion, which caused local delamination of the coating (Figure 5a). This effect was significantly reduced by polishing the substrates prior to the coating deposition process (Figure 5b). This observation confirms the fact that reducing the roughness and removing various defects of the substrate as a result of polishing contributes to a reduction in the number of gaps and grooves in the coating, thus improving its corrosion resistance. However, the reduction of pitting corrosion by polishing the substrate was not observed for the reference coating in which a chromium (Cr) coating was used as an interlayer (Figure 6a,b). This may suggest that in the Cr/Cr_2_N system, there may be an increase in the number of local corrosion centers on the interface sample/corrosive environment due to mechanisms other than just coating defects. Dong et al. [22] and Cunha et al. [26] indicate that one of the reasons for this phenomenon may be the large difference between the surface of the existing coating defects, i.e., pores, gaps, or pinholes, which form the cathodic area, and their inner surface, on the border with the substrate or the interlayer, which form the anodic area, which may significantly deteriorates the corrosion properties of ceramic coatings. This type of mechanism could be the cause of the observed larger number of pitting corrosion points in the case of the Cr/Cr_2_N coating (Figure 5c).

SEM microscopy revealed one more property of S-phase/Cr_2_N composites. Namely, during the corrosion tests, the process had two stages. In the first stage, the outer Cr_2_N coating was completely dissolved, and then the corrosion processes affected the S-phase interlayer itself. Moreover, the S-phase interlayer is still present on almost the entire surface of the area exposed to the corrosive environment at the final stage of the test, already with a high positive corrosion potential (Figure 5a,b). A similar effect was observed in the case of coatings with a chromium interlayer. However, pitting corrosion centers caused simultaneous dissolution of both the outer Cr_2_N coating and the Cr interlayer (Figure 5c).

However, in the case of the S-phase/Cr_2_N composite coatings, additional polishing of the substrate prior to the deposition of the coating visibly improves the resistance of the coating to pitting corrosion (Figure 6c–h). This indicates a better corrosion resistance of coatings with an S-phase interlayer compared to coatings with a chromium interlayer.

**Figure 7:**
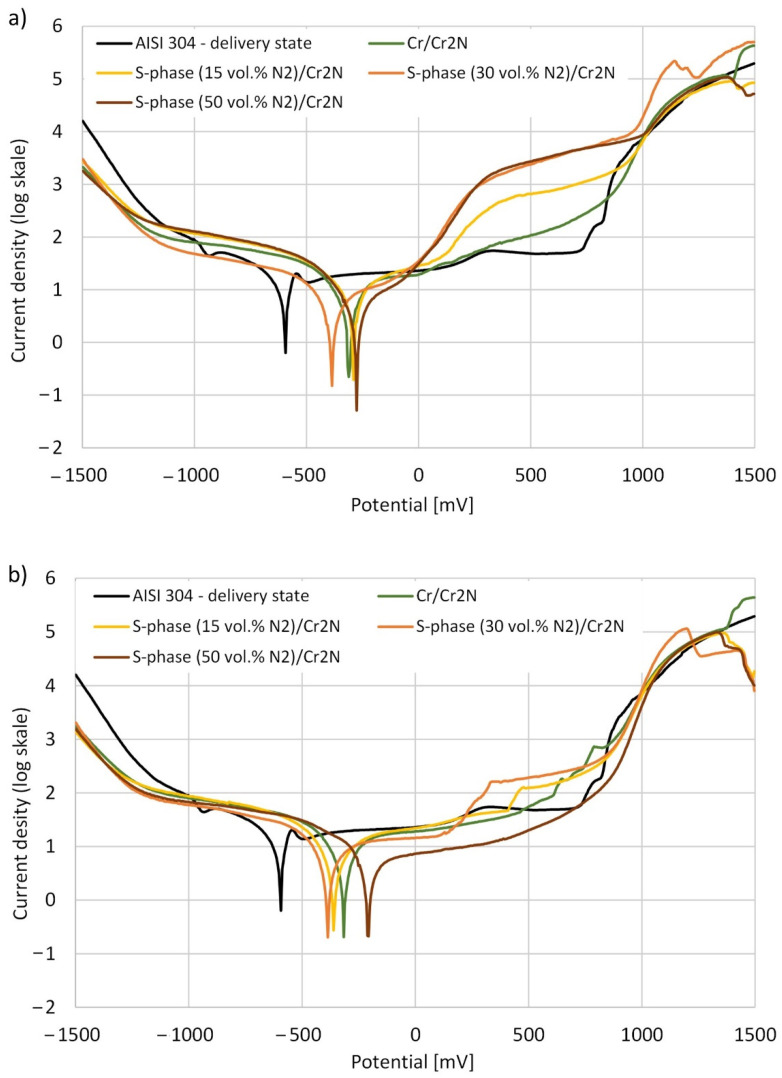
Polarization curves for composite coatings deposited on: (**a**) substrate in as delivery state and (**b**) polished substrate.

The polarization curves of composite coatings are shown in Figure 7. Their analysis by means of the Tafel method allowed to determine the corrosion potential of the coatings (E_corr_), the corrosion current density (i_corr_), and the polarization resistance of the coatings (R_p_). Average values of two measurements for each of the tested samples are summarized in Table 2.

The corrosion potential of all deposited composite coatings was between −400 and −200 mV and was higher (less negative) in comparison to the corrosion potential of uncoated austenitic stainless steel, for which it was −576 mV. There was no significant difference in the corrosion potential of the coatings depending on the substrate initial state. Moreover, the corrosion current density for the tested coatings was lower compared to the uncoated austenitic stainless steel. It should be noted that the lowest value of the corrosion current density was measured for the Cr/Cr_2_N coating and it was about 2 µA/cm^2^. However, for S-phase/Cr_2_N composite coatings and for uncoated austenitic stainless steel it was 3–6 μA/cm^2^ and about 10 µA/cm^2^, respectively. The values of the corrosion potential and the corrosion current density were similar, while the polarization resistance was higher for the tested coatings compared to the values for the Cr/CrN_x_ systems noted in the literature [33].

The polarization curves for the coatings deposited on the substrates in as delivery state show two plateau ranges (Figure 7a), while for coatings deposited on polished substrates, this effect is much less visible or there is only one area of passive range (Figure 7b). In terms of corrosion properties, the wider the plateau range in the passive state, the greater the corrosion resistance of the material. In the case of coatings deposited on substrates in as delivery state, the two passive plateau ranges may first correspond to the passivation of the outer Cr_2_N coating, which becomes completely dissolved as the polarization voltage increases (Figure 5), followed by passivation and dissolving of the interlayer. Moreover, the increase in the corrosion current density observed in the plateau range indicates that the corrosion processes are initiated by coating defects. A similar effect was observed by Kim Y.S. et al. [45] for the niobium coatings deposited by magnetron sputtering on the austenitic stainless steel 316 L. This proves that by reducing the number of coating defects by polishing the substrate prior to the coating deposition process, it is possible to increase the resistance of the coating to pitting and crevice corrosion.

**Figure 8:**
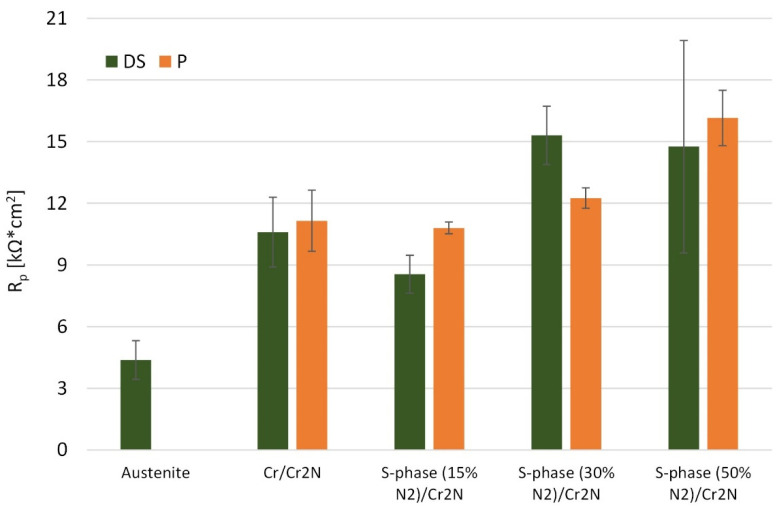
The influence of interlayer type on the polarization resistance R_p_ for investigated composite coatings.

As shown in Table 1, the polarization resistance R_p_ for composite coatings with S-phase interlayer has values similar to that of the Cr/Cr_2_N coating. An exception is the coating with an S-phase interlayer deposited at a 15% vol. of nitrogen in the working atmosphere, for which the polarization resistance of a coating deposited on a substrate in as delivery state was slightly lower compared to the other coatings. It was also observed that the increase in nitrogen content in the working atmosphere during the deposition of the S-phase interlayer, resulting in an increase in nitrogen content in the interlayer itself, increased the polarization resistance of the S-phase/Cr_2_N composite coating (Figure 8). This effect may be related to the improvement in the corrosion properties of the S-phase with the increase in nitrogen content, which was observed by Fossati et al. [42]. The results of the tests carried out indicate that the increase in nitrogen content in the S-phase interlayer improves not only the corrosion resistance of the interlayer itself, but also of the entire S-phase/Cr_2_N composite coating. It should also be noted that the polarization resistance R_p_ of all produced composite coatings was higher compared to the uncoated austenitic stainless steel and corresponded to the values reported in the literature for coatings made of chromium nitride [28,33].

## 4. Conclusions

All deposited composite coatings increased the corrosion resistance of the austenitic stainless steel. Electrochemical potentials of Cr/Cr_2_N and S-phase/Cr_2_N composite coatings were comparable and in both cases they were higher than for the austenitic stainless steel. The corrosion current density value for S-phase/Cr_2_N composite coatings was lower than that for the austenitic stainless steel, but higher than that of the Cr/Cr_2_N coating. The S-phase/Cr_2_N composite coatings for which the nitrogen content in the S-phase was the highest showed the highest polarization resistance. In contrast, the S-phase/Cr_2_N coatings with a smaller amount of nitrogen in the S-phase showed slightly lower polarization resistance, with values similar to those measured for the Cr/Cr_2_N coating. However, it was still higher than for austenitic stainless steel. The polarization curves for the coatings deposited on the substrates in as delivery state showed the effect of the two-stage plateau range. This effect was much weaker for coatings deposited on polished substrates. In addition, the coatings on the polished substrates showed a wider passive range compared to the coatings on the substrates in as delivery state. This was most likely the result of a two-step dissolving process, first of the outer Cr_2_N coating, followed by the interlayers. Pitting corrosion was mainly associated with defects in the coating, and their number was significantly reduced by the process of polishing the substrates prior to the deposition process. A significant reduction in the number of corrosion centers was also achieved by using the S-phase as an interlayer in place of the chromium interlayer. The conducted research showed that S-phase can be effectively used as an interlayer for the deposition of chromium nitride coatings.

## Figures and Tables

**Table 1:** Coatings deposition conditions.

Outer Coating	Interlayer	Nitrogen Concentration during Interlayer Doposition	Substrate
Cr_2_N	chrome	0	1× DS + 1× P
	S-phase	15 vol.%	1× DS + 1× P
		30 vol.%	1× DS + 1× P
		50 vol.%	1× DS + 1× P

where: DS—substrate in delivery state; P—polished substrate.

**Table 2:** Corrosion potential (E_corr_), corrosion current density (i_corr_) and polarization resistance (R_p_) for investigated coatings.

	E_corr_ [mV]		i_corr_ [μA/cm^2^]		R_p_ [kΩ*cm^2^]	
Substrate initial state	DS	P	DS	P	DS	P
Austenitic stainless steel	−576		10.4		4.4	
Cr/Cr_2_N	−307	−317	1.5	2.1	10.6	11.2
S-phase (15 vol.% of N_2_)/Cr_2_N	−288	−355	4.5	5.1	8.6	10.8
S-phase (30 vol.% of N_2_)/Cr_2_N	−385	−378	6.1	5.1	15.3	12.3
S-phase (50 vol.% of N_2_)/Cr_2_N	−270	−202	3.1	4.2	14.8	16.2

## Data Availability

Data are available on request at corresponding authors.
